# Melatonin or Ramelteon for Delirium Prevention in the Intensive Care Unit: A Systematic Review and Meta-Analysis of Randomized Controlled Trials

**DOI:** 10.3390/jcm12020435

**Published:** 2023-01-05

**Authors:** Giuseppe Aiello, Micol Cuocina, Luigi La Via, Simone Messina, Giuseppe A. Attaguile, Giuseppina Cantarella, Filippo Sanfilippo, Renato Bernardini

**Affiliations:** 1 Department Biomedical and Biotechnological Sciences (BIOMETEC), Section of Pharmacology, University of Catania, 95123 Catania, Italy; 2 Department of Anesthesiology and Intensive Care, AOU “Policlinico-San Marco”, 95123 Catania, Italy; 3 School of Specialization in Anesthesiology and Intensive Care, University “Magna Graecia”, 88100 Catanzaro, Italy; 4 Clinical Toxicology Unit, University Hospital of Catania, 95123 Catania, Italy

**Keywords:** critical care, agonist, length of stay, mechanical ventilation, mortality

## Abstract

Melatonin modulates the circadian rhythm and has been studied as a preventive measure against the development of delirium in hospitalized patients. Such an effect may be more evident in patients admitted to the ICU, but findings from the literature are conflicting. We conducted a systematic review and meta-analysis of randomized controlled trials (RCTs). We assessed whether melatonin or ramelteon (melatonin agonist) reduce delirium incidence as compared to a placebo in ICU patients. Secondary outcomes were ICU length of stay, duration of mechanical ventilation (MV) and mortality. Estimates are presented as risk ratio (RR) or mean differences (MD) with 95% confidence interval (CI). Nine RCTs were included, six of them reporting delirium incidence. Neither melatonin nor ramelteon reduced delirium incidence (RR 0.76 (0.54, 1.07), *p* = 0.12; I^2^ = 64%), although a sensitivity analysis conducted adding other four studies showed a reduction in the risk of delirium (RR = 0.67 (95%CI 0.48, 0.92), *p* = 0.01; I^2^ = 67). Among the secondary outcomes, we found a trend towards a reduction in the duration of MV (MD −2.80 (−6.06, 0.47), *p* = 0.09; I^2^ = 94%) but no differences in ICU-LOS (MD −0.26 (95%CI −0.89, 0.37), *p* = 0.42; I^2^ = 75%) and mortality (RR = 0.85 (95%CI 0.63, 1.15), *p* = 0.30; I^2^ = 0%). Melatonin and ramelteon do not seem to reduce delirium incidence in ICU patients but evidence is weak. More studies are needed to confirm this finding.

## 1. Introduction

Delirium is defined as an acute disorder of attention and of global cognitive functions [1]. It is a very common condition in hospitalized patients, and its incidence grows further in the ICU setting, where it may reach up to 80% prevalence [2]. This is not surprising since patients admitted to the ICU are, on average, older and sicker than other hospitalized patients in other wards. Notably, patients experiencing an episode of delirium seem to have a higher risk of increased short- and long-term mortality, worse cognitive function and an increased length of hospital stay [2]. However, these results are still debated [3].

Among the causes of the development of delirium in ICU patients, a major role is played by the alteration of the circadian rhythm, which is caused by a number of factors such as very frequent exposure to light and noises due to alarms. These factors determine the fragmentation of the sleep architecture [4]. Moreover, many sedative drugs, such as benzodiazepines, may be a trigger for delirium in ICU patients [2]. Therefore, the most recent guidelines suggest avoiding benzodiazepines to treat psycho-motor agitation in ICU patients [3]

The management of delirium in ICUs is mainly focused on non-pharmacological modalities (early mobilization, limiting the use of sedative drugs and methods to improve the quality of sleep [5]) as no drug has shown clear advantages, although from pharmacological perspectives, several drugs have been investigated. Among antipsychotic drugs, haloperidol, a typical first-generation antipsychotic, has been the most extensively studied in the treatment of delirium. The evidence of its efficacy seems limited [6], as its administration did not show significant benefits on the incidence of delirium, mortality and length of stay in ICU as compared with a placebo [5]. These findings have also been confirmed by a recent meta-analysis [7]. Olanzapine and quetiapine may represent other pharmacological alternatives, but they are associated with the risk of adverse events (i.e., metabolic abnormalities [8] and QT-c prolongation [9]). Due to this risk profile and the lack of evidence of benefits in the prevention and/or treatment of delirium [10], these second-generation antipsychotics have not received significant clinical implementation. Conversely, melatonin has generated significant interest. It is a hormone produced in the pineal gland that positively modulates the circadian rhythm. Since the dysregulation of the circadian rhythm appears to be one of the major causes of delirium [11], melatonin could be a valid preventive measure to avoid the development of delirium in hospitalized patients. Moreover, melatonin is not expensive and has other pleiotropic physiological actions, including antioxidant and anti-inflammatory effects, which could be advantageous in ICU patients [12,13].

The results of studies investigating the use of melatonin for delirium prevention in hospitalized patients are conflicting, depending possibly on the type of patients and on the setting (ICU vs. non-ICU). Despite some meta-analyses having suggested advantages of melatonin in the ICU setting [14], a more recent multi-center randomized controlled trial (RCT) failed to demonstrate a reduction in the risk of delirium with the use of melatonin [15]. Therefore, in order to investigate the usefulness of melatonin or another melatonin agonist (i.e., ramelteon) for delirium prevention in the ICU setting, we conducted a systematic review and meta-analysis focusing on results of RCTs.

## 2. Methods

### 2.1. Search Strategy and Selection Criteria

We conducted a systematic search and meta-analysis of RCTs comparing melatonin or its agonist ramelteon (at any dosage) with placebos in ICU patients.

The protocol was registered on the PROSPERO database (CRD 42022320435). The meta-analysis was conducted according to the PRISMA guidelines [16], and a PRISMA checklist is provided in the Supplementary Materials. Studies were included according to the PICOS approach (Supplementary Materials). For the primary analysis, we considered only RCTs including critically ill patients admitted to the ICU, treated with melatonin and/or its agonist ramelteon at any dosage compared to a placebo or no other treatment. The primary outcome was delirium incidence, while secondary outcomes included days of mechanical ventilation, length of stay in ICU and mortality. Studies on the pediatric population and those where melatonin (or ramelteon) was administered before ICU admission were excluded. Non-randomized prospective and retrospective clinical studies were considered for sensitivity analysis only. We included only manuscripts published in English. A computerized search of the MEDLINE (PubMed) and EMBASE databases was conducted from inception until 1 April 2022 to identify relevant articles. EMBASE findings were included only if they were published not later than 4 years (to allow for a peer-review process and publication).

Our core search combined two groups of terms. The first one included the terms “melatonin” OR “melatonin agonist” OR “ramelteon”, whilst the second group included the words “delirium” or “agitation” or “length of stay” or “ventilation”. Study selection for determining eligibility for inclusion in the systematic review and data extraction was performed independently by two reviewers (G.A. and M.C.) with the supervision of another author (L.L.V.). Discordances were resolved by involving senior authors (F.S., R.B. and G.C.). A manual search was conducted independently by four authors (F.S., L.L.V., M.C. and G.A.) to explore the reference lists for the findings of the systematic search.

### 2.2. Statistical Analysis

The meta-analysis was conducted using the Cochrane Review Manager version 5.4 (The Cochrane Collaboration, London, United Kingdom). Dichotomous outcomes were analyzed as risk ratio (RR) with 95% confidence interval (CI) using the Mantel–Haenszel method. Continuous outcomes were analyzed as mean difference (MD) with 95% CI, and p-values were considered significant if <0.05. Heterogeneity across studies was estimated by I^2^. Due to high statistical heterogeneity, a random effect model was used. Potential publication bias was assessed by inspection of the funnel plot (Supplementary Materials).

### 2.3. Quality Assessment

The methodological quality of the included RCTs was evaluated using the Cochrane RoB2 tool, which incorporates the following domains: randomization process, deviation from intended interventions, missing outcome data, measurement of the outcome and selection of the reported result.

Grading of the evidence was performed according to the recommendations of the Grading of Recommendations Assessment, Development and Evaluation working group by three authors (G.A., M.C. and F.S.) using the GRADEpro software available at https://gdt.gradepro.org/ (accessed on 27 September 2022).

### 2.4. Outcomes

We primarily compared the reported efficacy of melatonin (or ramelteon) in the prevention of delirium in the ICU. Secondary endpoints evaluated were the duration of mechanical ventilation (MV), the ICU length of stay (LOS) and mortality at the longest follow-up reported.

### 2.5. Subgroup and Sensitivity Analyses and Trial Sequential Analysis (TSA)

Subgroup analyses were considered according to the type of melatonin agonist used. Due to heterogeneity in the retrieved studies, we could not perform subgroup analyses according to the characteristics of the ICU population or their admission diagnosis.

Sensitivity analyses were performed including results from non-randomized prospective and retrospective clinical studies and from abstracts, as well as results from studies including a mixed population with patients from the ICU and other acute wards.

We performed a TSA on the primary outcome using the TSA Software (Copenhagen Trial Unit’s TSA Software^®^; Copenhagen, Denmark). The “information size” (sample size needed to achieve robust findings) was computed assuming an alpha risk of 5% with a power of 80%, using a random effect model and the relative risk reduction gathered from the results of the forest plot. Further details on the TSA and its interpretation are available elsewhere [17,18].

## 3. Results

Our systematic search identified 302 studies via PubMed and 441 via EMBASE. Two extra studies were retrieved manually; therefore, a total of 745 abstracts were screened. Of these, 709 were excluded due to not being focused on the topic of interest. One article was excluded because the authors did not report the incidence of delirium but rather the median change in Richmond Agitation–Sedation Scale score [19], which is not a scale usually adopted for delirium assessment. After title and abstract selection, only 13 studies [4,15,20,21,22,23,24,25,26,27,28,29,30] were judged to be of potential interest for our quantitative analyses. However, when considering the study design as per the PICOS criteria, we included only nine RCTs in the primary analysis [4,15,20,22,23,25,26,28,29], whilst one RCT [24], two retrospective studies [21,27] and one recent conference abstract [30] (RCT) were used for sensitivity analyses only.

The characteristics of the included studies are shown in Table 1. Of the nine RCTs included in the primary analysis, the most recent one enrolled 841 patients and was by far the largest of all those included. Eight RCTs randomized patients to melatonin or placebo groups, whilst the remaining one used ramelteon in the intervention group. The overall results of our meta-analysis are shown in Table 2.

### 3.1. Primary Outcome

**Figure 1:**
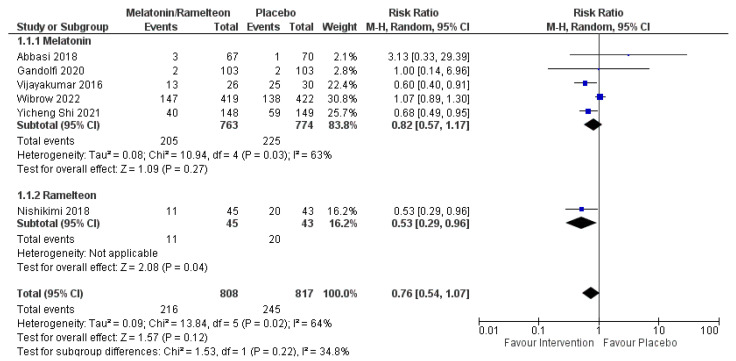
Forest plot reporting the differences in delirium incidence in patients admitted in the intensive care unit and treated with melatonin or ramelteon as compared to a placebo. Abbreviations: CI, confidence interval; M-H, Mantel–Haenszel. (Abbasi et al. [20]), (Gandolfi et al. [23]), (Vijayakumar et al. [22]), (Wibrow et al. [15]), (Yicheng Shi [29]), (Nishikimi et al. [25]).

Delirium incidence: As shown in Figure 1, six RCTs compared melatonin/ramelteon vs. placebo in the prevention of delirium in ICUs, including 1625 patients. Treatment with melatonin/ramelteon did not lower the risk of delirium (RR = 0.76 (95%CI 0.54, 1.07), *p* = 0.12, I^2^ = 64%). The subgroup analysis according to the treatment drug (melatonin or ramelteon) did not show differences (*p* = 0.22).

A sensitivity analysis was conducted adding four studies—namely, two retrospective studies, one RCT where the population included ICU patients together with others admitted in acute wards and one RCT published as a conference abstract. In this analysis, treatment with melatonin/ramelteon was associated with a statistically significant reduction in the risk of delirium (RR = 0.67 (95%CI 0.48, 0.92), *p* = 0.01; I^2^ = 67%) without subgroup differences (*p* = 0.28).

### 3.2. Secondary Outcomes

Days of MV: Four RCTs, reporting data on 989 patients, showed a trend in favor of treatment with melatonin with a lower number of days spent on MV (MD −2.80 (95%CI −6.06, 0.47), *p* = 0.09; I^2^ = 94%; Supplementary Materials). The sensitivity analysis conducted with the retrospective studies added showed significant differences between groups, favoring melatonin (MD −1.46 (95%CI −2.56, −0.35), *p* = 0.01; I^2^ = 91%).

ICU-LOS: Eight RCTs reported data on ICU-LOS (*n* = 1453 patients) with no difference between groups (MD −0.26 (95%CI −0.89, 0.37), *p* = 0.42; I^2^ = 75%) and in the subgroup analysis (*p* = 0.81; Supplementary Materials). The results of the sensitivity analysis including two retrospective studies (1843 patients in total) did not change (MD 0.03 [95%CI −0.59, 0.66], *p* = 0.92; I^2^ = 83%).

Mortality: Seven RCTs reported mortality, with data on 1661 patients. Melatonin/ramelteon treatment was not associated with differences in mortality (RR = 0.85 (95%CI 0.63, 1.15), *p* = 0.30; I^2^ = 0%), with no differences between subgroups (Supplementary Materials). In the sensitivity analysis, we added one retrospective study, and the results showed no differences in mortality (RR = 0.82 (95%CI 0.63, 1.06), *p* = 0.13; I^2^ = 0%).

### 3.3. Risk of Bias Assessments and Publication Bias

The results of the assessment of risk of bias according to the RoB2 tool are reported in the Supplementary Materials. In particular, in terms of the overall evaluation of the risk of bias, three RCTs were deemed to be at high risk, three had some concerns and only four were classified as low-risk RCTs. The visual inspection of the funnel plots (Supplementary Materials) showed no risk of publication bias.

### 3.4. GRADE of Evidence and TSA

The results of assessment of the GRADE of evidence for the primary and secondary outcomes are reported in Table 3. Due to the serious rating mainly in terms of risk of bias and indirectness of findings, the outcomes investigated were judged to have a very low level of certainty.

**Figure 2:**
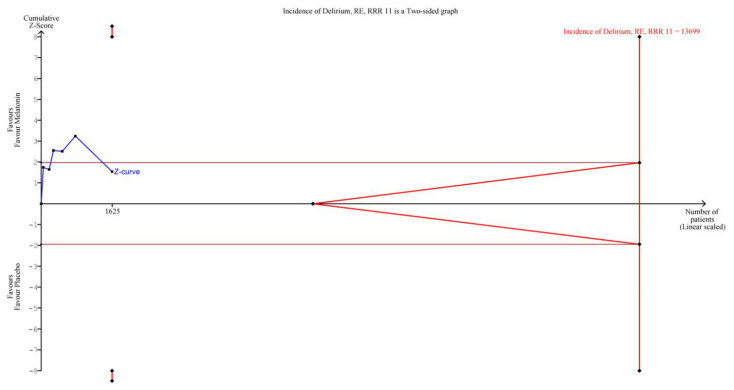
Trial sequential analysis on delirium incidence. Abbreviations: RE, random effect; RRR, relative risk reduction.

The TSA for the primary outcome (Figure 2) showed that the results are not yet robust and more research is warranted. In particular, considering a relative risk reduction of 11%, the Z-curve crossed neither the conventional boundary nor the futility boundary, with a ratio of patients recruited/needed of *n* = 1.625/13.699. We also performed a TSA assuming a doubled relative risk reduction (22%), and also in this case, the result suggested that more research is needed and the findings are not robust (ratio of patients recruited/needed = 1.625/7.417).

## 4. Discussion

In this meta-analysis of RCTs, we primarily assessed the usefulness of melatonin (or ramelteon, a melatonin agonist) for delirium prevention in the ICU setting. We found that melatonin and ramelteon as compared to a placebo did not reduce the incidence of delirium, nor did they improve any of the results regarding the secondary outcomes (days of MV, ICU-LOS and mortality). However, it must be considered that our results do not seem definitive for several reasons. First, the TSA showed that the information size required was far from being reached, with 1625 patients enrolled in the studied RCTs, representing 8.5% of the sample needed (*n* = 13,699). Even after artificially doubling the effect size estimation (relative risk reduction of 22%), the TSA still suggested that a bigger information size is needed (*n* = 7417) and more studies are warranted; therefore, the current results on the efficacy of melatonin or ramelteon in reducing the risk of delirium in the ICU setting cannot be considered robust. Second, the GRADE of evidence suggests a very low certainty of the evidence due to the risk of bias in several studies and the indirectness of the findings. Third, the primary findings were changed by the sensitivity analysis conducted adding two retrospective studies, one RCT where the population included patients admitted to the ICU together with others from acute wards and another RCT that is currently reported only as a conference abstract. Indeed, this analysis suggested that treatment with melatonin/ramelteon is associated with a statistically significant reduction in the risk of delirium in ICU patients (RR = 0.67, *p* = 0.01).

Our results are consistent with those of the largest multi-center RCT conducted in 12 Australian hospitals, where the use of melatonin was not associated with an increase in the proportion of delirium-free assessments in ICU patients; moreover, the findings of this RCT were confirmed across all subgroups according to diagnostic category, age and baseline delirium risk. In this RCT, the secondary outcomes also were not different between patients treated with melatonin or a placebo.

The idea to use melatonin in critically ill patients is based on the greater risk of development of delirium in this population of patients due to the greater severity of their clinical conditions and the characteristics of the ICU in terms of the noisy environment and alteration of the day–night cycle [32,33,34]. There is evidence of very low melatonin levels in critically ill patients [33,35,36,37,38,39,40]; thus, it is theoretically reasonable to expect greater effects of melatonin in this setting.

From the pharmacological perspective, some considerations should be made. Receptors for melatonin (MT) are found in different tissues in the central nervous system and periphery. In the brain, MT_1_ and MT_2_ receptors are located on neuronal membranes in the suprachiasmatic nucleus of the hypothalamus, an area associated with circadian rhythms. Activation of the MT_1_ receptor induces sleep, while stimulation of the MT_2_ receptor appears to be related to the light–dark synchronization of the biological clock [41]. Melatonin has been studied for numerous indications, and it is currently mainly administered to prevent jet lag and to improve the onset, duration and quality of sleep (at a dose of 0.5–5 mg before going to sleep, with a maximum dosage of 10-20 mg) [42,43]. Moreover, when administered before anesthesia, it may reduce preoperative anxiety in adults, being as effective as midazolam [44]. Clinical studies also support the decrease in postoperative anxiety after melatonin administration [44]. Meanwhile, ramelteon (selective MT_1_–MT_2_ agonist) has been approved for the pharmacological treatment of insomnia [45] and appears to be more effective as a hypnotic agent. Notably, as ramelteon is metabolized by the CYP1A2 and CYP2C9 isoforms of cytochrome P450, the drug should not be used in combination with inhibitors of CYP1A2 (i.e., ciprofloxacin) or CYP2C9 (i.e., fluconazole), and it should be administered with caution in patients with hepatic insufficiency. Moreover, in the presence of a CYP inducer such as rifampicin, the plasma levels of both ramelteon and its active metabolite may be significantly reduced [46,47]. We found no differences in the subgroup analyses, although it must be considered that only one study investigated the use of ramelteon, and thus, the number of patients is rather small and does not allow to draw meaningful conclusions on differences between the two agonists.

Regarding delirium prevention, melatonin administration has also been investigated outside the ICU setting. One of the largest studies conducted by de Jonghe et al. [48] evaluated over 450 patients undergoing acute hip surgery and reported no differences in delirium with a dose of 3 mg/day of melatonin compared to a placebo for 5 days [48]. Whether melatonin may provide a benefit if included in a series of bundle and preventative strategies [49,50,51,52] that also include non-pharmacological interventions such as frequent reorientation, early mobilization, exposure to sunlight and sleep hygiene [51,53,54,55,56,57] needs to be defined.

One of the issues with our meta-analysis is that we included studies from different ICU populations, ranging from non-selected cohorts to very selected categories of patients such as those with intracranial hemorrhage or with intoxication from organophosphorus compounds. Additionally, the lack of significant differences in the incidence of delirium might be partially explained by the low mean age of patients in most of the studies included, since the probability of developing delirium increases by 2% per year after age 65 [58].

Another issue that could have affected the results of this meta-analysis is the different scales used to assess delirium in each study included (e.g., Confusion Assessment Method Intensive Care Unit (CAM-ICU) and Intensive Care Delirium Screening Checklist (ICDSC)). Moreover, the scales were administered with variable time intervals between studies since delirium is considered a fluctuating rather than a continuous disorder. The dose of melatonin used by the included RCTs was also very different, and in the studies included in the analysis of delirium, it ranged from 3 to 10 mg/day; furthermore, the duration of administration varied from 5 days to 2 weeks. Notably, slow-release formulations have been studied [24,25], and a novel dosing regimen with a loading dose followed by supplemental smaller doses has been described and may be considered for further investigation [31].

We also included one study that administered a melatonin dose of 30 mg/day. However, this study did not report the effects on delirium but rather focused on secondary outcomes only. Another issue is that in the patients with a short ICU-LOS, melatonin (or ramelteon) treatment may have had no time to provide benefits on delirium and/or on sleep quality, as it would have needed to be continued into the ward environment.

## 5. Conclusions

Our meta-analysis of randomized controlled trials suggests that melatonin and ramelteon, as compared to placebos, do not significantly reduce the incidence of delirium in the ICU setting. Moreover, melatonin and ramelteon do not seem to reduce the duration of mechanical ventilation during ICU stay or influence mortality. However, the findings have very low certainty of evidence and more research is warranted.

## Figures and Tables

**Table 1:** Characteristics of the populations and the interventions in the included studies selected for meta-analysis. Studies are divided into primary and sensitivity analyses.

Studies	Outcomes Reported	Participants	Intervention	Sample	Age (Mean)	Severity Score APACHE II	Dosage
Primary analysis							
Wibrow, 2022 Int Care Med [15]	Delirium, MV, LOS, mortality	ICU	Melatonin	419	61.9	17.3	4 mg/day (2 weeks or LOS)
			Placebo	422	61.9	17.5	
Shi, 2021 Heart Surg Forum [29]	Delirium, mortality	ICU after PCI	Melatonin	148	71.5	**	3 mg/day for 1 week
			Placebo	149	71.6	**	
Gandolfi, 2020 Crit. Care Med. [23]	Delirium, LOS, mortality	ICU	Melatonin	102	60.0	1.5 *	10 mg/day for 7 days
			Placebo	101	57.0	1.42 *	
Abbasi,2018 Iran J Pharm Res [20]	Delirium, LOS, mortality	mixed ICU surgical/medical	Melatonin	67	52.5	8.1	3 mg/day for 5 days
			Placebo	70	49.9	7.3	
Nishikimi, 2018 Crit Care Med [25]	Delirium, LOS, mortality	medical ICU	Ramelteon	45	67.0	23.98	8 mg/day
			Placebo	43	66.5	23.95	
Vijayakumar, 2016 Indian J Anaesth. [22]	Delirium, MV, LOS	ICU poisoning with organophosphorus	Melatonin	26	36.9	10.2	3 mg/day
			Placebo	30	38	8.56	
Bellapart, 2020 Crit Care Res Pract [31]	LOS	ICU	Melatonin	21	54.75	21.5	6 mg/day
			Placebo	12	57.25	22.5	
Soltani, 2020 Eur J Trauma Emerg Surg [28]	MV, LOS, mortality	ICU for intracranial hemorrhage	Melatonin	26	34.62	7.45	3 mg/day
			Placebo	26	36.85	8.14	
Dianatkhah, 2017 J Res Pharm Pract [26]	MV, LOS, mortality	ICU for hemorrhagic stroke	Melatonin	20	57.7	17.6	30 mg/day
			Placebo	20	52.9	16.9	
**Sensitivity analysis**							
Bandyopadhya, 2021 ESICM Lives 2021 [30]	Delirium	ICU	Melatonin	54	/	/	3 mg/day
			Placebo	54	/	/	
Romero, 2021 Pharmacotherapy [27]	Delirium, MV, LOS	ICU	Melatonin/ramelteon	131	65.51	4 *	3 mg/day melatonin
			Placebo	27	60.7	4.125 *	8 mg/day ramelteon
Baumgartner, 2019 Pharmacotherapy [21]	Delirium, MV, LOS, mortality	Mixed or cardiac ICU medical/surgical	Melatonin	117	60.5	17.5	3.5 mg/day (1–10 mg range)
			Placebo	115	59.5	15.75	
Hatta, 2014 JAMA Psychiatry [24]	Delirium	Medical ICU and acute wards	Ramelteon	33	78.3	14.6	8 mg/day
			Placebo	34	78.2	13.5	

* Mean SOFA score. ** Missing data. Abbreviations: APACHE, Acute Physiologic Assessment and Chronic Health Evaluation II; MV, mechanical ventilation (days); ICU, intensive care unit; PCI, percutaneous coronary intervention; LOS, length of stay; SOFA, Sequential Organ Failure Assessment.

**Table 2:** Summary of the results of the primary and secondary outcomes comparing treatment with melatonin/ramelteon vs. placebo (control group).

Outcome	Studies	Patients (*n*)	RR or MD (95% CI)	*p*-Value	Heterogeneity	
					I^2^	*p*-Value
Delirium incidence	6	1625	RR 0.76 (0.54, 1.07)	0.12	64%	0.02
Days of MV	4	989	MD −2.80 (−6.06, 0.47)	0.09	94%	<0.00001
Mortality	7	1661	RR 0.84 (0.62, 1.13)	0.26	0%	0.60
LOS in ICU	8	1453	MD −0.26 (−0.89, 0.37)	0.42	75%	0.0002

Abbreviations: CI, confidence interval; ICU, intensive care unit; LOS, length of stay; MD, mean difference; MV, mechanical ventilation; RR, relative risk.

**Table 3:** Evaluation of quality of evidence according to Grading of Recommendations Assessment, Development and Evaluation (GRADE) working group.

Melatonin or Ramelteon as Compared to Placebo for Prevention of Delirium in the Intensive Care Unit											
Certainty Assessment							Summary of Findings				
Participants (Studies)	Risk of Bias	Inconsistency	Indirectness	Imprecision	Publication Bias	Overall Certainty of Evidence	Study Event Rates (%)		Relative Effect (95% CI)	Anticipated Absolute Effects	
							Placebo	Melatonin or Ramelteon		Risk with Placebo	Risk Difference with Melatonin or Ramelteon
Delirium incidence											
1625 (6 RCTs)	Serious	Not serious	Very serious ^a^	Not serious	None	Very low	245/817 (30.0%)	216/808 (26.7%)	RR 0.76 (0.54 to 1.07)	300 per 1000	72 fewer per 1000 (from 138 fewer to 21 more)
**Days of mechanical ventilation**											
989 (4 RCTs)	Serious	Not serious	Very serious	Serious ^b^	None	Very low	498	491	-	The mean duration of mechanical ventilation was **0** days	MD 2.8 lower (6.06 lower to 0.47 higher)
**Mortality at longest follow-up**											
1661 (7 RCTs)	Serious	Not serious	Very serious	Serious ^c^	None	Very low	85/833 (10.2%)	71/828 (8.6%)	RR 0.84 (0.62 to 1.13)	102 per 1000	16 fewer per 1000 (from 39 fewer to 13 more)
**Length of stay in the intensive care unit**											
1453 (8 RCTs)	Very serious	Not serious	Very serious	Serious ^d^	None	Very low	726	727	-	The mean length of stay was 0 days	MD 0.26 lower (0.89 lower to 0.37 higher)

Explanations: ^a^. Despite the fact that these are data from patients in intensive care, there is still a certain heterogeneity in the study populations, such that it cannot be said that the evidence is completely direct. ^b^. The imprecision in the data is due to the approximation for certain studies that provide data as medians and interquartile ranges rather than means and standard deviations. ^c^. The timing of mortality is not entirely clear (ICU, 28-day or longer timeframe). ^d^. Some data are reported in days and others in hours, requiring an adjustment. Abbreviations: CI, confidence interval; MD, mean difference; RCT, randomized clinical trial; RR, risk ratio.

## Supplementary Materials

The following supporting information can be downloaded at: https://www.mdpi.com/article/10.3390/jcm12020435/s1, Figure S1: PRISMA flowchart graphically describes the process of screening, selection and inclusion of articles; Table S1: PICOS approach for selecting clinical studies in the systematic search and meta-analysis; Figure S2: funnel plot of primary outcome—delirium incidence—in primary analysis to evaluate the existence of publication bias; Figure S3: forest plot of secondary outcome reporting the differences of days of MV in patients admitted in the intensive care unit and treated with Melatonin or Ramelteon as compared to Placebo; Figure S4: funnel plot of secondary outcome—days of MV—in primary analysis to evaluate the existence of publication bias; Figure S5: forest plot of secondary outcome reporting the differences of length of stay in patients admitted in the intensive care unit and treated with Melatonin or Ramelteon as compared to Placebo; Figure S6: funnel plot of secondary outcome—length of stay—in primary analysis to evaluate the existence of publication bias; Figure S7: forest plot of secondary outcome reporting the differences of mortality in patients admitted in the intensive care unit and treated with Melatonin or Ramelteon as compared to Placebo; Figure S8: funnel plot of secondary outcome—mortality—to evaluate the existence of publication bias; Figure S9: forest plot of primary outcome in sensitivity analysis reporting the differences of delirium incidence in patients admitted in the intensive care unit and treated with Melatonin or Ramelteon as compared to Placebo; Figure S10: funnel plot of primary outcome—delirium incidence—in sensitivity analysis to evaluate the existence of publication bias; Figure S11: forest plot of secondary outcome in sensitivity analysis reporting the differences of days of MV in patients admitted in the intensive care unit and treated with Melatonin or Ramelteon as compared to Placebo; Figure S12: funnel plot of secondary outcome—days of MV—in sensitivity analysis to evaluate the existence of publication bias; Figure S13: forest plot of secondary outcome in sensitivity analysis reporting the differences of length of stay in patients admitted in the intensive care unit and treated with Melatonin or Ramelteon as compared to Placebo; Figure S14: funnel plot of secondary outcome—length of stay—in sensitivity analysis to evaluate the existence of publication bias; Figure S15: forest plot of secondary outcome in sensitivity analysis reporting the differences of mortality in patients admitted in the intensive care unit and treated with Melatonin or Ramelteon as compared to Placebo; Figure S16: funnel plot of secondary outcome—mortality—in sensitivity analysis to evaluate the existence of publication bias; Table S2: risk of bias of RCTs evaluated in primary analysis.
