# Supplementary material for: Melatonin or Ramelteon for Delirium Prevention in the Intensive Care Unit: A Systematic Review and Meta-Analysis of Randomized Controlled Trials
Source: J Clin Med. 2023 Jan 5;12(2):435. doi: 10.3390/jcm12020435 (PMC9863078; doi:10.3390/jcm12020435)
Supplement: Supplementary file 1 [file jcm-12-00435-s001.zip › jcm-2057125-supplementary.pdf]

Melatonin or Ramelteon for Delirium Prevention in the Intensive Care Unit: a Systematic Review and Meta-Analysis of Randomized Controlled Trials

Supplementary

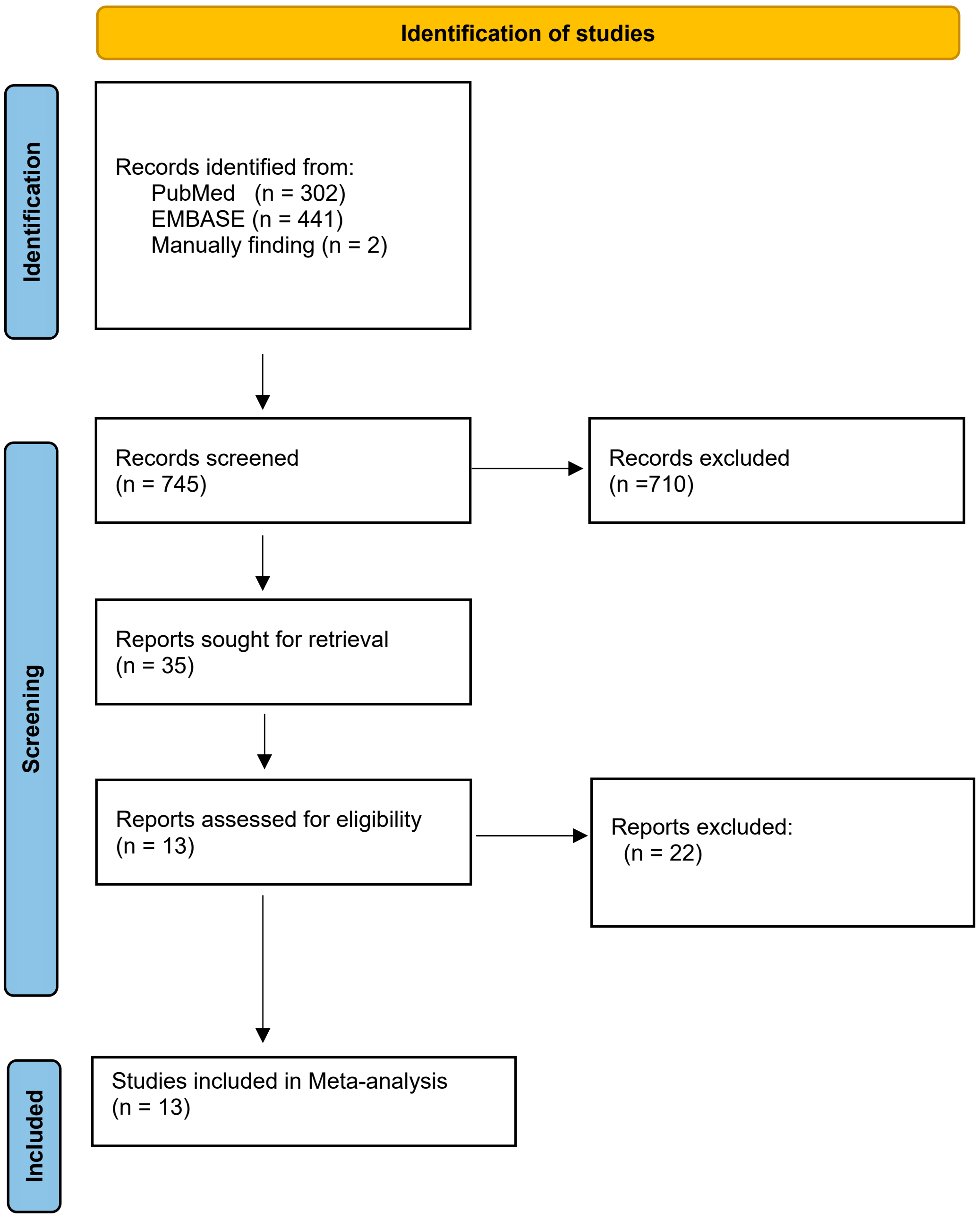

Figure S1. PRISMA flowchart graphically describes the process of screening, selection and inclusion of articles. Abbreviation. PRISMA: Preferred Reporting Items for Systematic reviews and Meta-Analyses

PICOS	
Participants	Critically ill patients admitted to ICU.
Intervention	Melatonin and/or its agonist (Ramelteon), at any dosage.
Comparison	Placebo or no other treatment.
Outcomes	Primary: incidence of Delirium.
	Secondary: days of mechanical ventilation, length of stay in ICU, mortality.
Study design	Randomized controlled trials (primary analysis).
	Non randomized prospective and retrospective studies (sensitivity analysis only).

Table S1. PICOS approach for selecting clinical studies in the systematic search and meta-analysis. Abbreviation ICU: intensive care unit; PICOS: population, intervention, comparison, outcome, study design

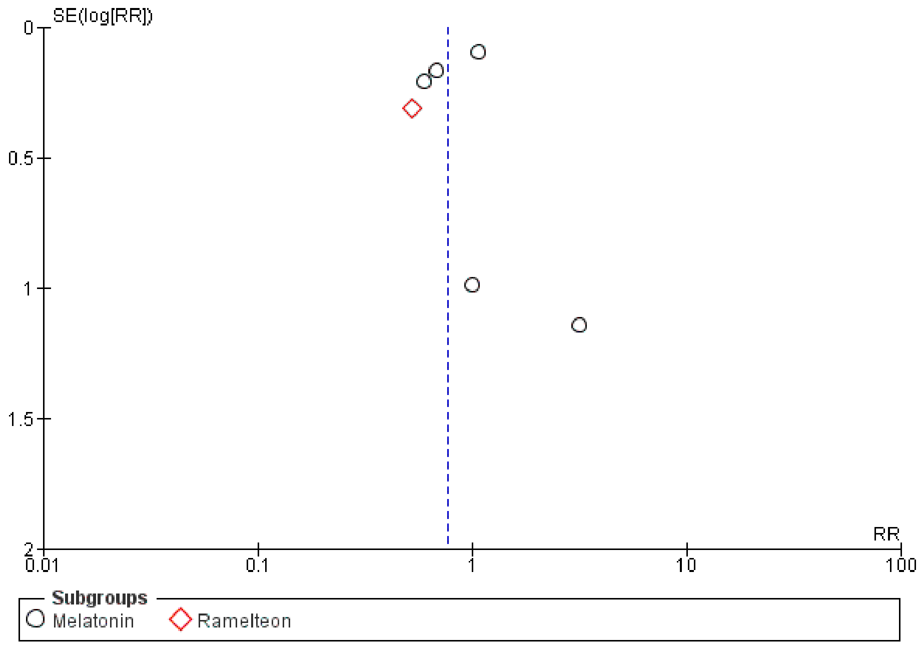

Figure S2. Funnel plot of primary outcome – delirium incidence – in primary analysis to evaluate the existence of publication bias.

Study or Subgroup	Melatonin Mean	Melatonin SD	Melatonin Total	Placebo Mean	Placebo SD	Placebo Total	Weight	Mean Difference IV, Random, 95% CI
**1.2.1 Melatonin RCTs**								
Dianatkhah 2017	6.5	4.06	20	12	4.62	20	23.2%	-5.50 [-8.20, -2.80]
Soltani 2020	7	3	26	12	4	26	25.2%	-5.00 [-6.92, -3.08]
Vijayakumar 2016	3.88	3.52	26	5.26	5.52	30	24.0%	-1.38 [-3.77, 1.01]
Wibrow 2022	1.08	0.99	419	0.82	0.79	422	27.6%	0.26 [0.14, 0.38]
**Subtotal (95% CI)**			**491**			**498**	**100.0%**	**-2.80 [-6.06, 0.47]**
Heterogeneity: Tau² = 10.06; Chi² = 47.77, df = 3 (P < 0.00001); I² = 94%								
Test for overall effect: Z = 1.68 (P = 0.09)								
**Total (95% CI)**			**491**			**498**	**100.0%**	**-2.80 [-6.06, 0.47]**
Heterogeneity: Tau² = 10.06; Chi² = 47.77, df = 3 (P < 0.00001); I² = 94%								
Test for overall effect: Z = 1.68 (P = 0.09)								
Test for subgroup differences: Not applicable								

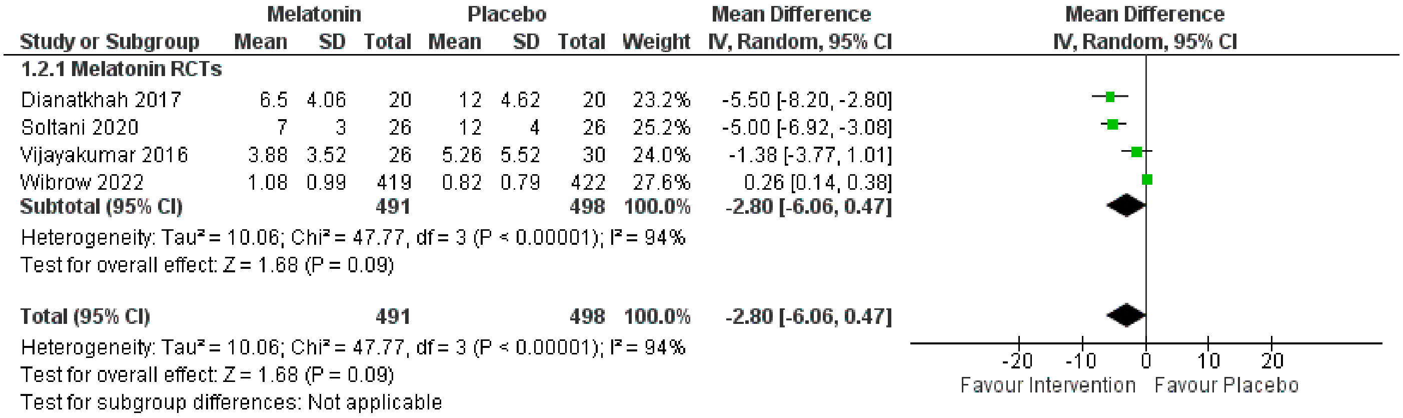

Figure S3. Forest plot of secondary outcome reporting the differences of days of MV in patients admitted in the intensive care unit and treated with Melatonin or Ramelteon as compared to Placebo. Abbreviation. CI: confidence interval; M-H: Mantel-Haenszel; MV: mechanical ventilation

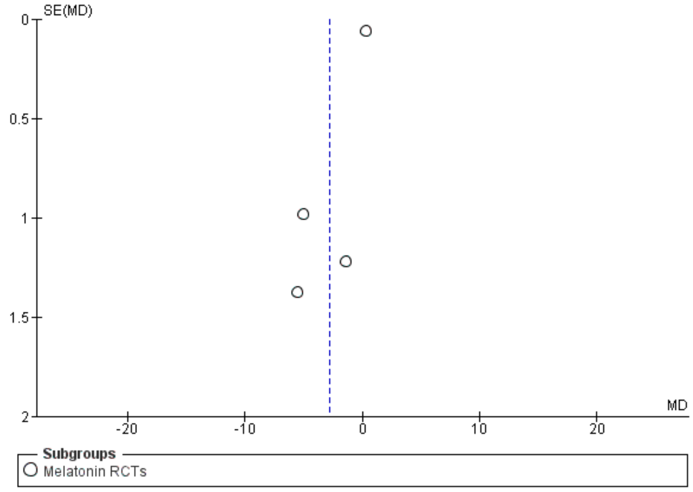

Figure S4. Funnel plot of secondary outcome – days of MV – in primary analysis to evaluate the existence of publication bias. Abbreviation. MV: mechanical ventilation.

Study or Subgroup	Melatonin/Ramelteon Mean	SD	Total	Placebo Mean	SD	Total	Weight	Mean Difference IV, Random, 95% CI
**1.4.1 Melatonin**								
Abbasi 2018	8.8	5.9	67	9.8	10.6	70	4.2%	-1.00 [-3.86, 1.86]
Bellapart 2020	25	5.78	21	24.25	5.49	12	2.3%	0.75 [-3.22, 4.72]
Dianatkhah 2017	10.75	4.35	20	14.25	4.93	20	4.2%	-3.50 [-6.38, -0.62]
Gandolfi 2020	5	2.33	103	5	2.33	103	23.2%	0.00 [-0.64, 0.64]
Soltani 2020	13.31	5.17	26	14.55	4.72	26	4.7%	-1.24 [-3.93, 1.45]
Vijayakumar 2016	7.65	3.58	26	9.36	6.35	30	4.8%	-1.71 [-4.37, 0.95]
Wibrow 2022	5.5	1.15	419	5	1.17	422	29.7%	0.50 [0.34, 0.66]
**Subtotal (95% CI)**			**682**			**683**	**73.1%**	**-0.26 [-1.03, 0.52]**
Heterogeneity: Tau² = 0.39; Chi² = 14.59, df = 6 (P = 0.02); I² = 59%								
Test for overall effect: Z = 0.65 (P = 0.52)								
**1.4.2 Ramelteon**								
Nishikimi 2018	1.5	1.02	45	1.86	0.92	43	26.9%	-0.36 [-0.77, 0.05]
**Subtotal (95% CI)**			**45**			**43**	**26.9%**	**-0.36 [-0.77, 0.05]**
Heterogeneity: Not applicable								
Test for overall effect: Z = 1.74 (P = 0.08)								
**Total (95% CI)**			**727**			**726**	**100.0%**	**-0.26 [-0.89, 0.37]**
Heterogeneity: Tau² = 0.35; Chi² = 27.86, df = 7 (P = 0.0002); I² = 75%								
Test for overall effect: Z = 0.80 (P = 0.42)								
Test for subgroup differences: Chi² = 0.06, df = 1 (P = 0.81), I² = 0%								

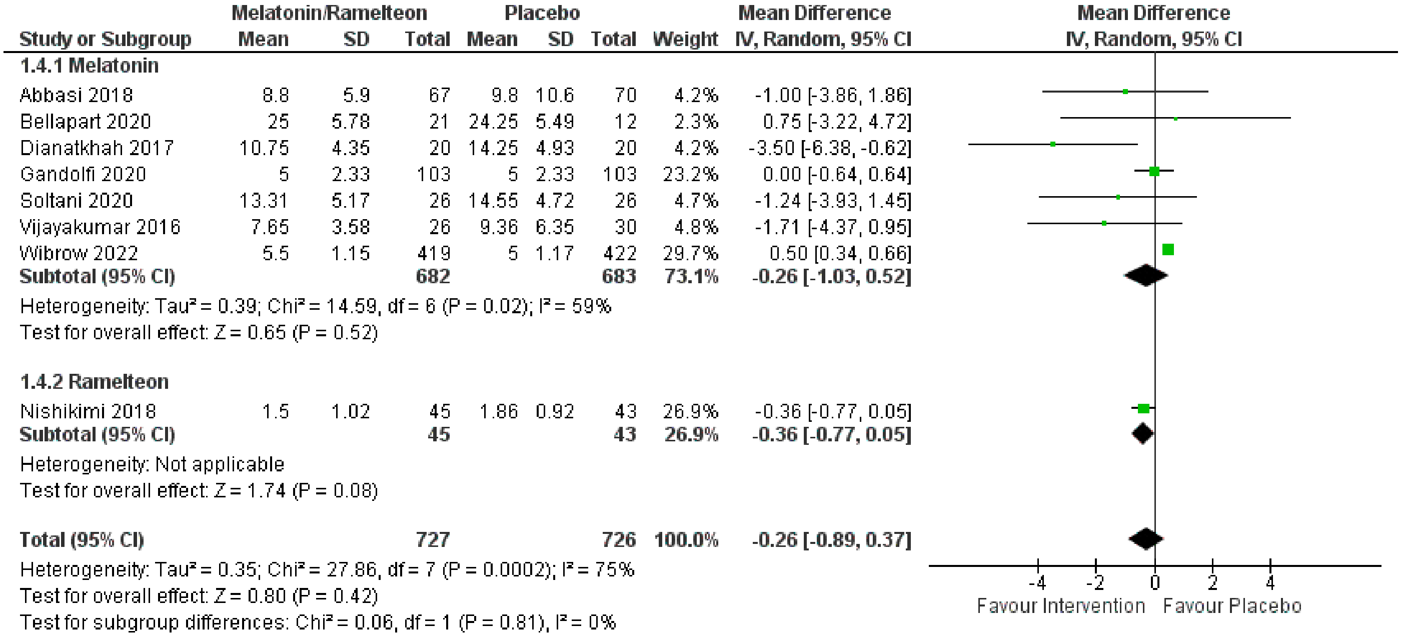

Figure S5. Forest plot of secondary outcome reporting the differences of length of stay in patients admitted in the intensive care unit and treated with Melatonin or Ramelteon as compared to Placebo. Abbreviation. CI: confidence interval; M-H: Mantel-Haenszel.

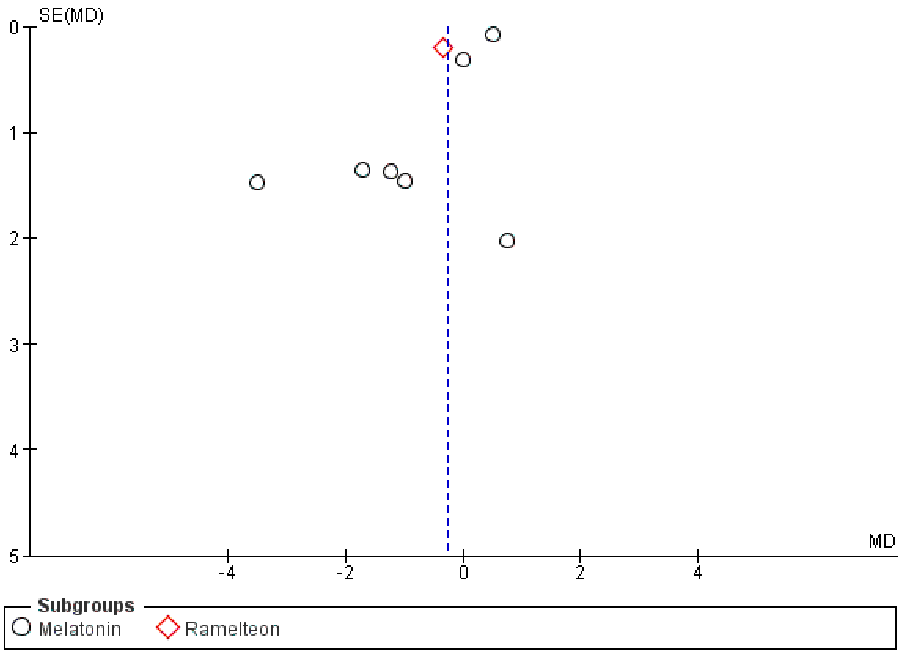

Figure S6. Funnel plot of secondary outcome –length of stay– in primary analysis to evaluate the existence of publication bias.

Study or Subgroup	Melatonin/Ramelteon Events	Melatonin/Ramelteon Total	Placebo Events	Placebo Total	Weight	Risk Ratio M-H, Random, 95% CI
**1.3.1 Melatonin RCTs**						
Abbasi 2018	9	67	7	70	10.6%	1.34 [0.53, 3.40]
Dianatkhah 2017	3	20	6	20	6.0%	0.50 [0.14, 1.73]
Gandolfi 2020	9	103	11	103	13.0%	0.82 [0.35, 1.89]
Soltani 2020	2	26	8	26	4.3%	0.25 [0.06, 1.07]
Wibrow 2022	27	419	29	422	35.7%	0.94 [0.57, 1.56]
Yicheng Shi 2021	18	148	21	149	26.6%	0.86 [0.48, 1.55]
**Subtotal (95% CI)**		**783**		**790**	**96.2%**	**0.85 [0.62, 1.15]**
Total events	68		82			
Heterogeneity: Tau² = 0.00; Chi² = 4.53, df = 5 (P = 0.48); I² = 0%						
Test for overall effect: Z = 1.05 (P = 0.29)						
**1.3.2 Ramelteon**						
Nishikimi 2018	3	45	3	43	3.8%	0.96 [0.20, 4.48]
**Subtotal (95% CI)**		**45**		**43**	**3.8%**	**0.96 [0.20, 4.48]**
Total events	3		3			
Heterogeneity: Not applicable						
Test for overall effect: Z = 0.06 (P = 0.95)						
**Total (95% CI)**		**828**		**833**	**100.0%**	**0.85 [0.63, 1.15]**
Total events	71		85			
Heterogeneity: Tau² = 0.00; Chi² = 4.55, df = 6 (P = 0.60); I² = 0%						
Test for overall effect: Z = 1.04 (P = 0.30)						
Test for subgroup differences: Chi² = 0.02, df = 1 (P = 0.88), I² = 0%						

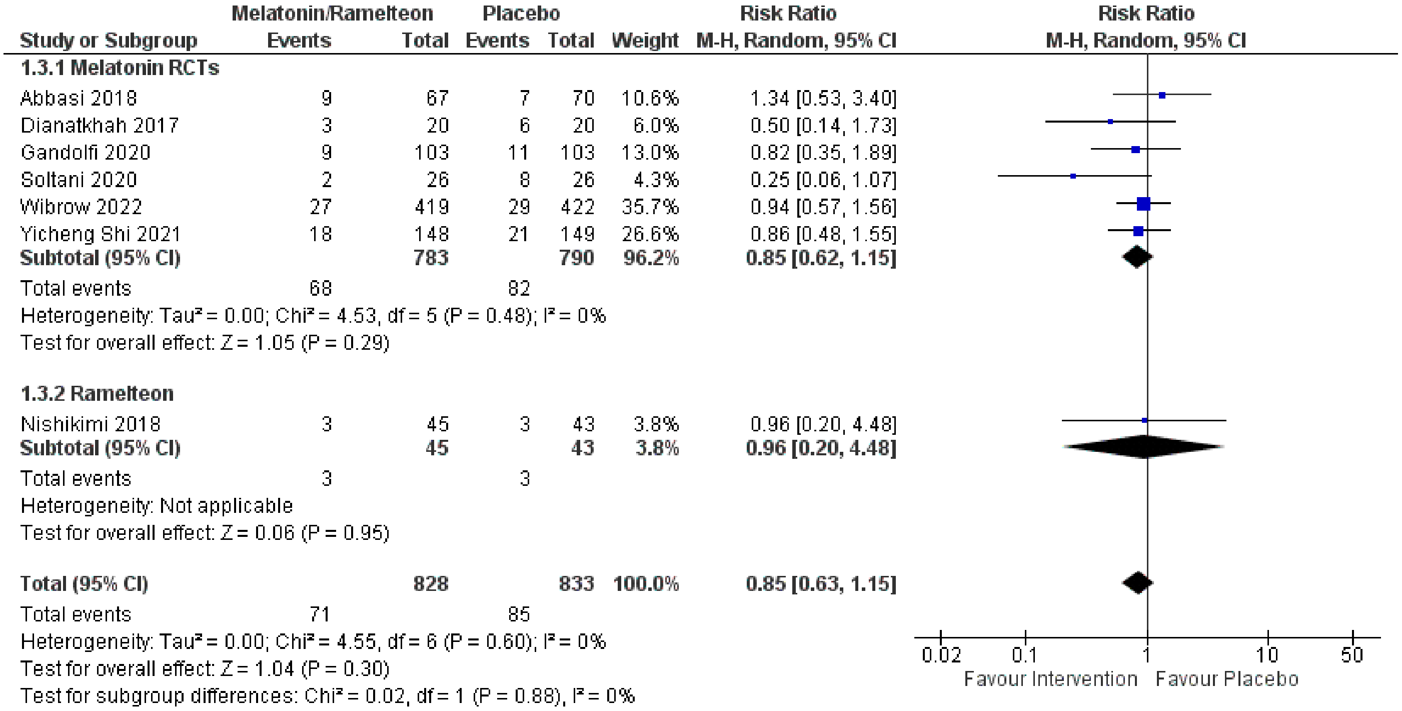

Figure S7. Forest plot of secondary outcome reporting the differences of mortality in patients admitted in the intensive care unit and treated with Melatonin or Ramelteon as compared to Placebo. Abbreviation. CI: confidence interval; M-H: Mantel-Haenszel.

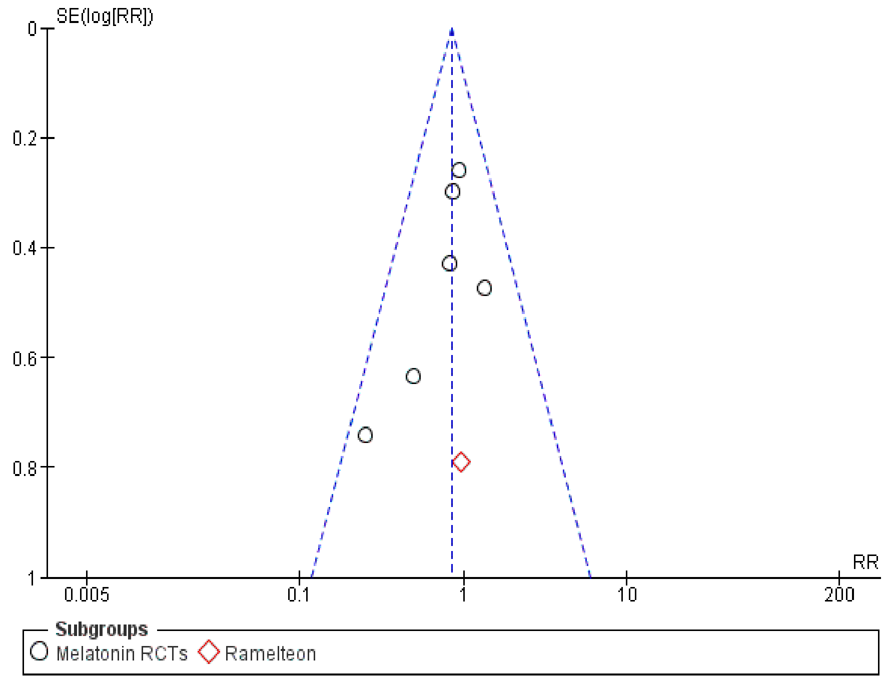

Figure S8. Funnel plot of secondary outcome – mortality – to evaluate the existence of publication bias.

Study or Subgroup	Melatonin/Ramelteon Events	Melatonin/Ramelteon Total	Placebo Events	Placebo Total	Weight	Risk Ratio M-H, Random, 95% CI
**2.1.1 Melatonin**						
Abbasi 2018	3	67	1	70	1.9%	3.13 [0.33, 29.39]
Gandolfi 2020	2	103	2	103	2.4%	1.00 [0.14, 6.96]
Vijayakumar 2016	13	26	25	30	15.1%	0.60 [0.40, 0.91]
Wibrow 2022	147	419	138	422	18.9%	1.07 [0.89, 1.30]
Yicheng Shi 2021	40	148	59	149	16.6%	0.68 [0.49, 0.95]
**Subtotal (95% CI)**		**763**		**774**	**54.9%**	**0.82 [0.57, 1.17]**
Total events	205		225			
Heterogeneity: $Tau^2 = 0.08$; $Chi^2 = 10.94$, df = 4 (P = 0.03); $I^2 = 63\%$						
Test for overall effect: Z = 1.09 (P = 0.27)						
**2.1.2 Ramelteon**						
Nishikimi 2018	11	45	20	43	11.8%	0.53 [0.29, 0.96]
**Subtotal (95% CI)**		**45**		**43**	**11.8%**	**0.53 [0.29, 0.96]**
Total events	11		20			
Heterogeneity: Not applicable						
Test for overall effect: Z = 2.08 (P = 0.04)						
**2.1.3 sensitivity**						
Bandyopadhyay 2021	16	54	25	54	13.6%	0.64 [0.39, 1.06]
Baumgartner 2019	9	117	28	115	10.3%	0.32 [0.16, 0.64]
Hatta 2014	1	33	11	34	2.3%	0.09 [0.01, 0.69]
Romero 2021	22	131	4	27	7.1%	1.13 [0.42, 3.02]
**Subtotal (95% CI)**		**335**		**230**	**33.3%**	**0.49 [0.24, 1.00]**
Total events	48		68			
Heterogeneity: $Tau^2 = 0.31$; $Chi^2 = 8.24$, df = 3 (P = 0.04); $I^2 = 64\%$						
Test for overall effect: Z = 1.96 (P = 0.05)						
**Total (95% CI)**		**1143**		**1047**	**100.0%**	**0.67 [0.48, 0.92]**
Total events	264		313			
Heterogeneity: $Tau^2 = 0.14$; $Chi^2 = 27.69$, df = 9 (P = 0.001); $I^2 = 67\%$						
Test for overall effect: Z = 2.47 (P = 0.01)						
Test for subgroup differences: $Chi^2 = 2.56$, df = 2 (P = 0.28), $I^2 = 21.8\%$						

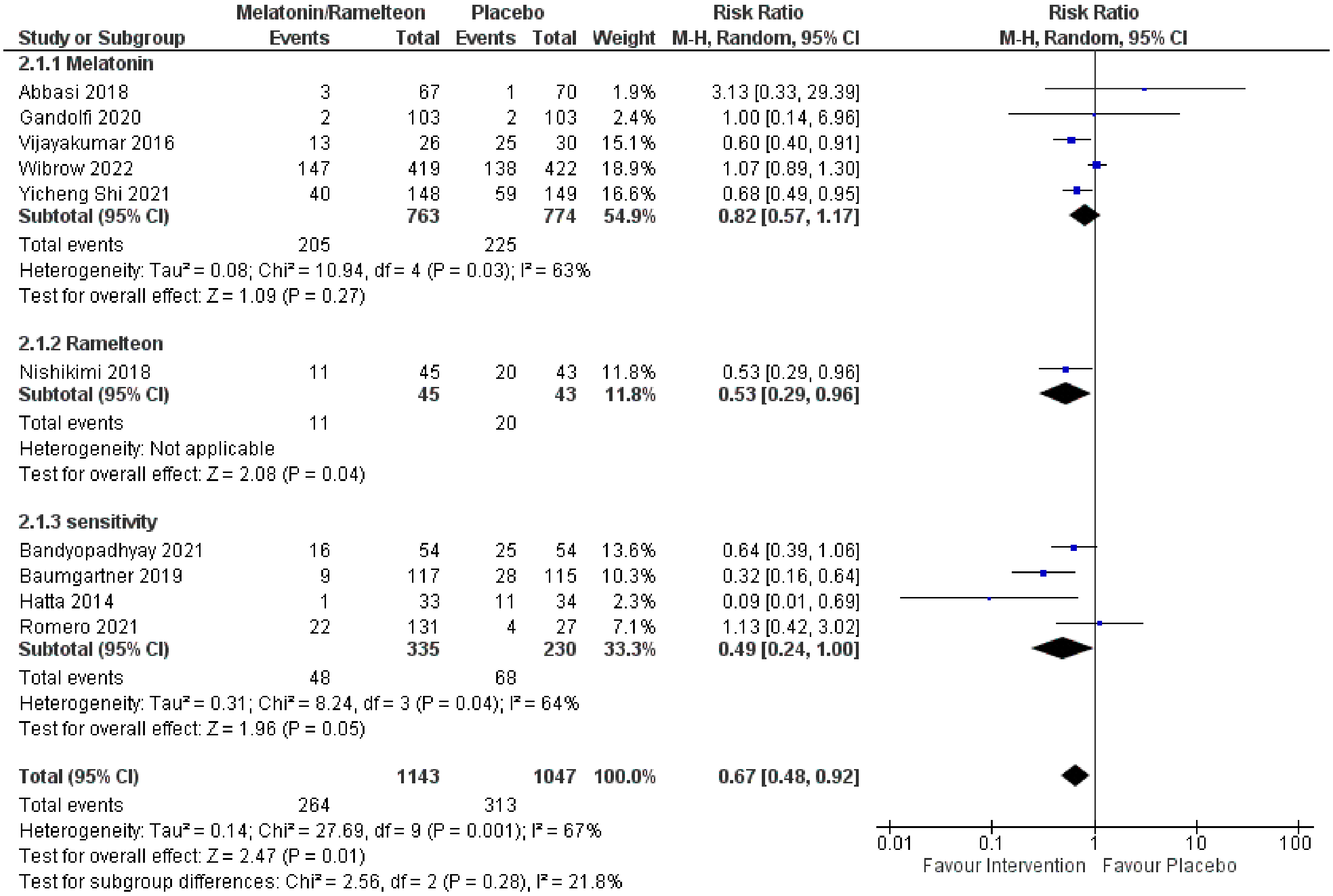

Figure S9. Forest plot of primary outcome in sensitivity analysis reporting the differences of delirium incidence in patients admitted in the intensive care unit and treated with Melatonin or Ramelteon as compared to Placebo. Abbreviation. CI: confidence interval; M-H: Mantel-Haenszel.

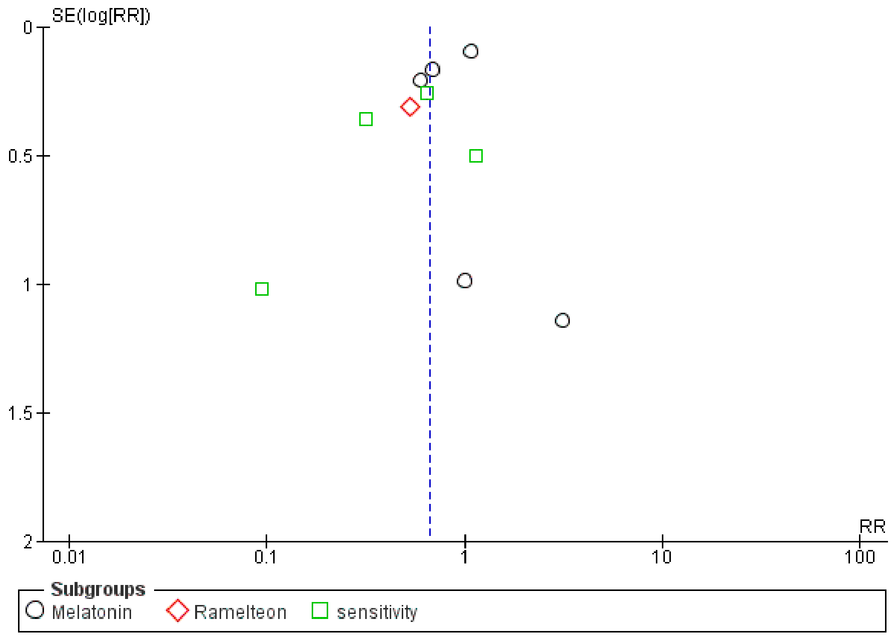

Figure S10. Funnel plot of primary outcome – delirium incidence – in sensitivity analysis to evaluate the existence of publication bias.

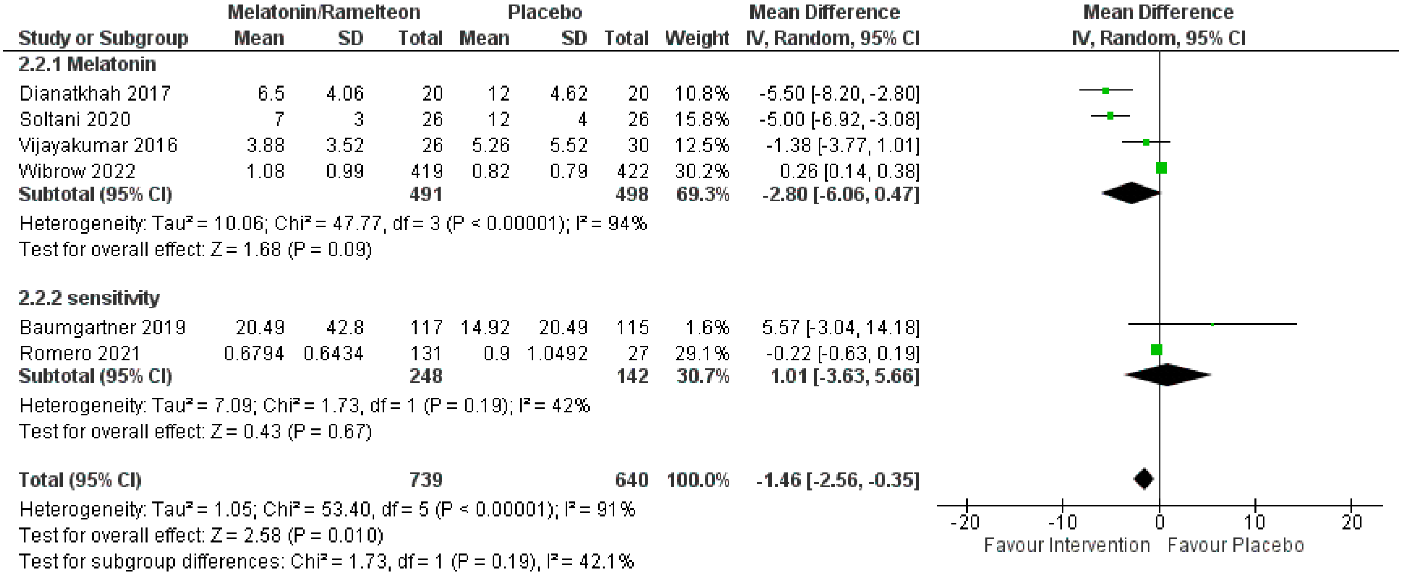

Figure S11. Forest plot of secondary outcome in sensitivity analysis reporting the differences of days of MV in patients admitted in the intensive care unit and treated with Melatonin or Ramelteon as compared to Placebo. Abbreviation. CI: confidence interval; M-H: Mantel-Haenszel; MV: mechanical ventilation.

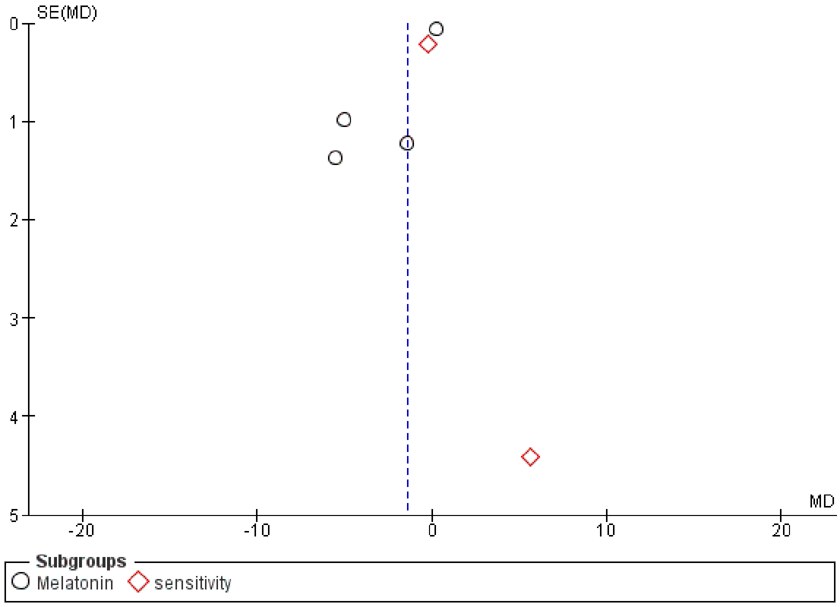

Figure S12. Funnel plot of secondary outcome – days of MV – in sensitivity analysis to evaluate the existence of publication bias. Abbreviation. MV: mechanical ventilation.

Study or Subgroup	Melatonin/Ramelteon Mean	SD	Total	Placebo Mean	SD	Total	Weight	Mean Difference IV, Random, 95% CI
**2.4.1 Melatonin**								
Abbasi 2018	8.8	5.9	67	9.8	10.6	70	3.9%	-1.00 [-3.86, 1.86]
Bellapart 2020	25	5.78	21	24.25	5.49	12	2.2%	0.75 [-3.22, 4.72]
Dianatkhah 2017	10.75	4.35	20	14.25	4.93	20	3.9%	-3.50 [-6.38, -0.62]
Gandolfi 2020	5	2.33	103	5	2.33	103	18.0%	0.00 [-0.64, 0.64]
Soltani 2020	13.31	5.17	26	14.55	4.72	26	4.3%	-1.24 [-3.93, 1.45]
Vijayakumar 2016	7.65	3.58	26	9.36	6.35	30	4.4%	-1.71 [-4.37, 0.95]
Wibrow 2022	5.5	1.15	419	5	1.17	422	21.9%	0.50 [0.34, 0.66]
**Subtotal (95% CI)**			**682**			**683**	**58.8%**	**-0.26 [-1.03, 0.52]**
Heterogeneity: Tau² = 0.39; Chi² = 14.59, df = 6 (P = 0.02); I² = 59%								
Test for overall effect: Z = 0.65 (P = 0.52)								
**2.4.2 Ramelteon**								
Nishikimi 2018	1.5	1.02	45	1.86	0.92	43	20.3%	-0.36 [-0.77, 0.05]
**Subtotal (95% CI)**			**45**			**43**	**20.3%**	**-0.36 [-0.77, 0.05]**
Heterogeneity: Not applicable								
Test for overall effect: Z = 1.74 (P = 0.08)								
**2.4.3 Sensitivity**								
Baumgartner 2019	15.6	30.5	117	17.1	37.3	115	0.5%	-1.50 [-10.28, 7.28]
Romero 2021	5.1752	1.1972	131	3.75	0.878	27	20.4%	1.43 [1.04, 1.81]
**Subtotal (95% CI)**			**248**			**142**	**20.9%**	**1.42 [1.03, 1.81]**
Heterogeneity: Tau² = 0.00; Chi² = 0.43, df = 1 (P = 0.51); I² = 0%								
Test for overall effect: Z = 7.15 (P < 0.00001)								
**Total (95% CI)**			**975**			**868**	**100.0%**	**0.03 [-0.59, 0.66]**
Heterogeneity: Tau² = 0.46; Chi² = 54.09, df = 9 (P < 0.00001); I² = 83%								
Test for overall effect: Z = 0.10 (P = 0.92)								
Test for subgroup differences: Chi² = 42.37, df = 2 (P < 0.00001), I² = 95.3%								

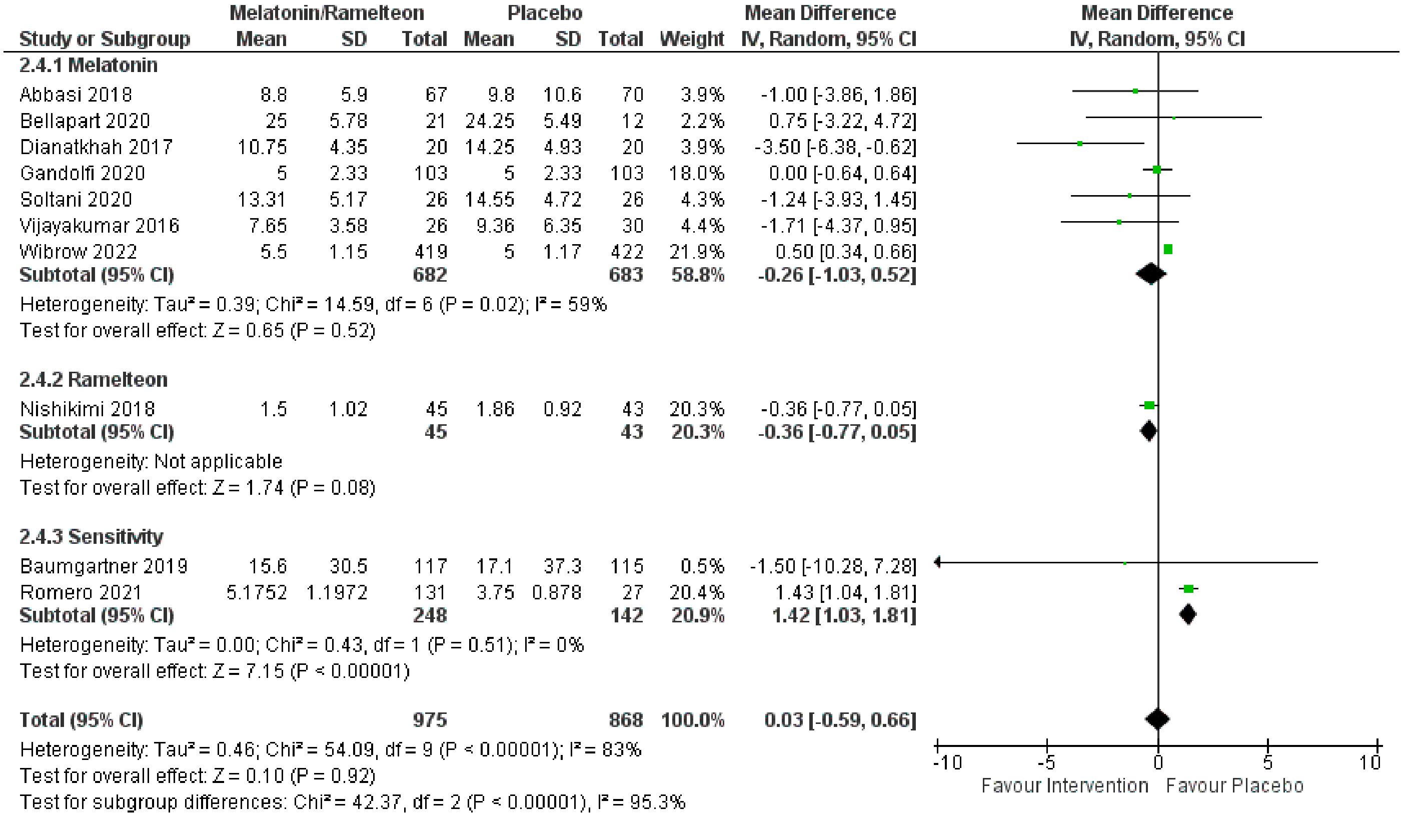

Figure S13. Forest plot of secondary outcome in sensitivity analysis reporting the differences of length of stay in patients admitted in the intensive care unit and treated with Melatonin or Ramelteon as compared to Placebo. Abbreviation. CI: confidence interval; M-H: Mantel-Haenszel.

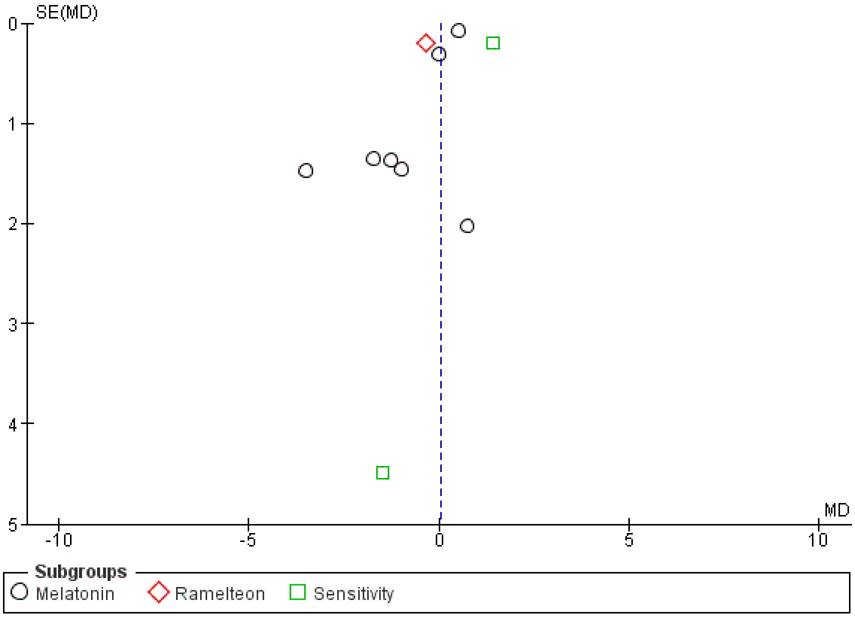

Figure S14. Funnel plot of secondary outcome – length of stay – in sensitivity analysis to evaluate the existence of publication bias.

Study or Subgroup	Melatonin/Ramelteon Events	Melatonin/Ramelteon Total	Placebo Events	Placebo Total	Weight	Risk Ratio M-H, Random, 95% CI
**2.3.1 Melatonin**						
Abbasi 2018	9	67	7	70	8.1%	1.34 [0.53, 3.40]
Dianatkhah 2017	3	20	6	20	4.6%	0.50 [0.14, 1.73]
Gandolfi 2020	9	103	11	103	10.0%	0.82 [0.35, 1.89]
Soltani 2020	2	26	8	26	3.3%	0.25 [0.06, 1.07]
Wibrow 2022	27	419	29	422	27.4%	0.94 [0.57, 1.56]
Yicheng Shi 2021	18	148	21	149	20.4%	0.86 [0.48, 1.55]
**Subtotal (95% CI)**		**783**		**790**	**73.7%**	**0.85 [0.62, 1.15]**
Total events	68		82			
Heterogeneity: $Tau^2 = 0.00$; $Chi^2 = 4.53$, df = 5 (P = 0.48); $I^2 = 0\%$						
Test for overall effect: Z = 1.05 (P = 0.29)						
**2.3.2 Ramelteon**						
Nishikimi 2018	3	45	3	43	2.9%	0.96 [0.20, 4.48]
**Subtotal (95% CI)**		**45**		**43**	**2.9%**	**0.96 [0.20, 4.48]**
Total events	3		3			
Heterogeneity: Not applicable						
Test for overall effect: Z = 0.06 (P = 0.95)						
**2.3.3 Sensitivity**						
Baumgartner 2019	18	117	25	115	23.3%	0.71 [0.41, 1.22]
**Subtotal (95% CI)**		**117**		**115**	**23.3%**	**0.71 [0.41, 1.22]**
Total events	18		25			
Heterogeneity: Not applicable						
Test for overall effect: Z = 1.24 (P = 0.22)						
**Total (95% CI)**		**945**		**948**	**100.0%**	**0.82 [0.63, 1.06]**
Total events	89		110			
Heterogeneity: $Tau^2 = 0.00$; $Chi^2 = 4.88$, df = 7 (P = 0.67); $I^2 = 0\%$						
Test for overall effect: Z = 1.51 (P = 0.13)						
Test for subgroup differences: $Chi^2 = 0.36$, df = 2 (P = 0.84), $I^2 = 0\%$						

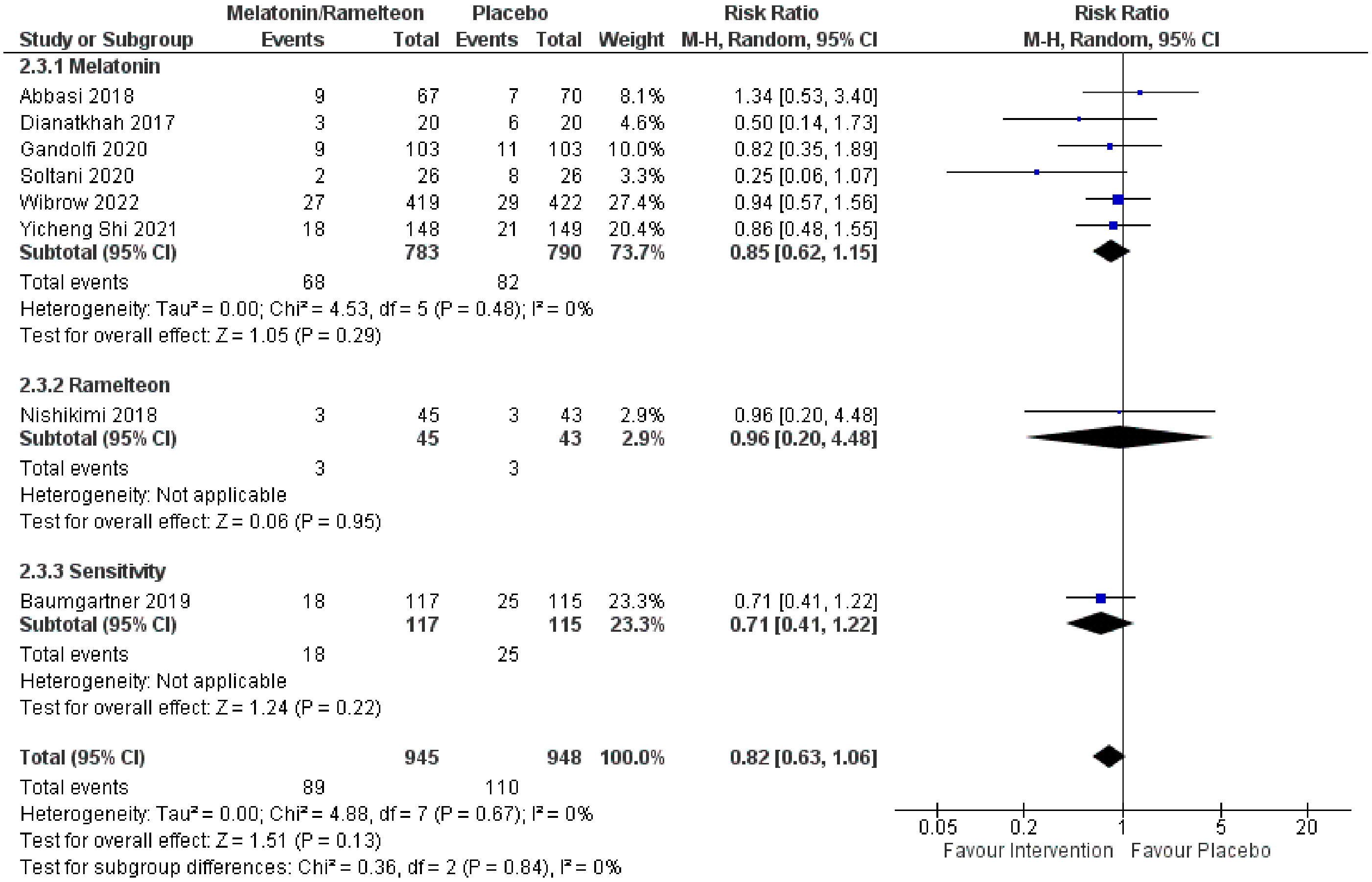

Figure S15. Forest plot of secondary outcome in sensitivity analysis reporting the differences of mortality in patients admitted in the intensive care unit and treated with Melatonin or Ramelteon as compared to Placebo. Abbreviation. CI: confidence interval; M-H: Mantel-Haenszel.

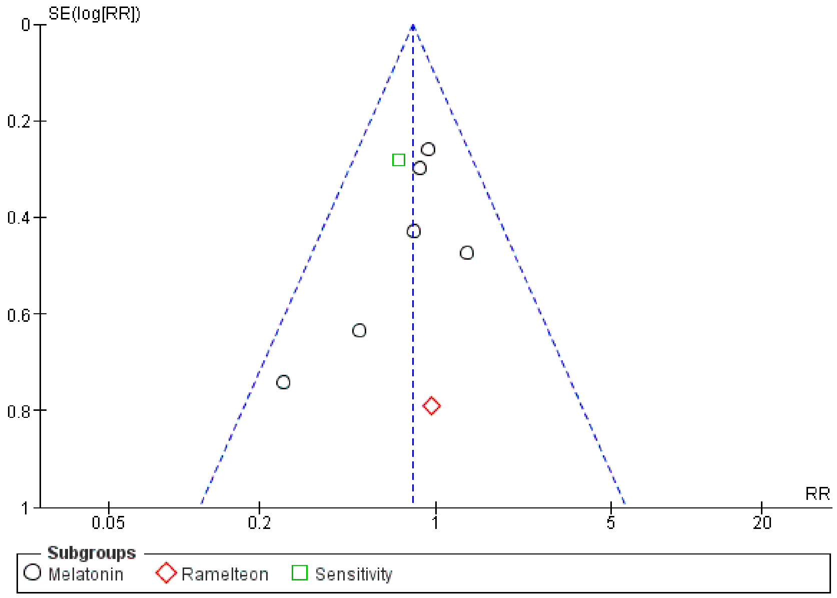

Figure S16. Funnel plot of secondary outcome – mortality – in sensitivity analysis to evaluate the existence of publication bias.

Risk of bias

Study ID	Experimental	Comparator	primary Outcome	Weight	D1	D2	D3	D4	D5	Overall
Abbasi 2018	Melatonin	Placebo	Delirium incidence	1	+	!	!	+	!	!
Bellapart 2020	Melatonin	Placebo	Incidence of delirium in ICU	1	+	+	-	+	+	-
Dianatkhah 2017	Melatonin	Placebo	Duration on mechanical ventilation and length of stay in ICU	1	!	!	+	-	!	-
Gandolfi 2020	Melatonin	Placebo	Delirium prevalence	1	!	!	+	+	!	!
Nishikimi 2018	Ramelteon	Placebo	Length of stay in ICU	1	+	+	+	+	!	+
Soltani 2020	Melatonin	Placebo	Mechanical ventilation - Length of stay in ICU	1	+	+	+	+	+	+
Vijayakumar 2016	Melatonin	Placebo	Duration of delirium in ICU	1	+	+	+	+	-	-
Wibrow 2022	Melatonin	Placebo	Delirium prevalence	1	+	+	+	+	+	+
Yicheng Shi 2021	Melatonin	Placebo	Delirium incidence after PCI	1	+	+	+	+	+	+

+ Low risk
! Some concerns
- High risk

D2 Deviations from the intended interventions
D3 Missing outcome data
D4 Measurement of the outcome
D5 Selection of the reported result

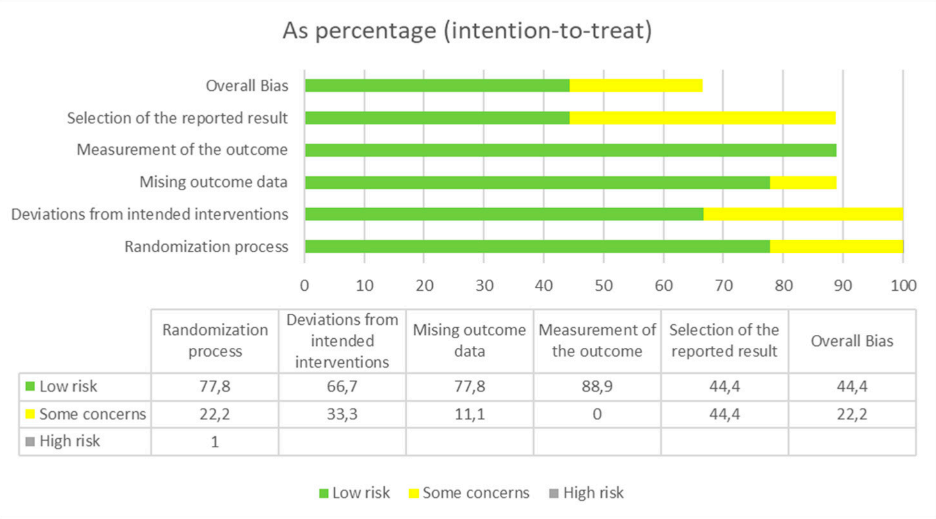

	Randomization process	Deviations from intended interventions	Mising outcome data	Measurement of the outcome	Selection of the reported result	Overall Bias
Low risk	77,8	66,7	77,8	88,9	44,4	44,4
Some concerns	22,2	33,3	11,1	0	44,4	22,2
High risk	1					

Table S2. Risk of bias of RCTs evaluated in primary analysis. Abbreviation. RCT: randomized clinical trial.

Melatonin or Ramelteon for delirium prevention in the intensive care unit: a systematic review and meta-analysis of randomized controlled trials.

PRISMA 2020 Checklist - Supplemental digital content

Section and Topic	Item #	Checklist item	Location where item is reported
**TITLE**			
Title	1	Identify the report as a systematic review.	Page 1
**ABSTRACT**			
Abstract	2	See the PRISMA 2020 for Abstracts checklist.	Page 2
**INTRODUCTION**			
Rationale	3	Describe the rationale for the review in the context of existing knowledge.	Page 3-4
Objectives	4	Provide an explicit statement of the objective(s) or question(s) the review addresses.	Page 4-5
**METHODS**			
Eligibility criteria	5	Specify the inclusion and exclusion criteria for the review and how studies were grouped for the syntheses.	Page 4-5
Information sources	6	Specify all databases, registers, websites, organisations, reference lists and other sources searched or consulted to identify studies. Specify the date when each source was last searched or consulted.	Page 5
Search strategy	7	Present the full search strategies for all databases, registers and websites, including any filters and limits used.	Page 5
Selection process	8	Specify the methods used to decide whether a study met the inclusion criteria of the review, including how many reviewers screened each record and each report retrieved, whether they worked independently, and if applicable, details of automation tools used in the process.	Page 5
Data collection process	9	Specify the methods used to collect data from reports, including how many reviewers collected data from each report, whether they worked independently, any processes for obtaining or confirming data from study investigators, and if applicable, details of automation tools used in the process.	Page 5
Data items	10a	List and define all outcomes for which data were sought. Specify whether all results that were compatible with each outcome domain in each study were sought (e.g. for all measures, time points, analyses), and if not, the methods used to decide which results to collect.	
	10b	List and define all other variables for which data were sought (e.g. participant and intervention characteristics, funding sources). Describe any assumptions made about any missing or unclear information.	
Study risk of bias assessment	11	Specify the methods used to assess risk of bias in the included studies, including details of the tool(s) used, how many reviewers assessed each study and whether they worked independently, and if applicable, details of automation tools used in the process.	Page 5
Effect measures	12	Specify for each outcome the effect measure(s) (e.g. risk ratio, mean difference) used in the synthesis or presentation of results.	Page 8-9

Melatonin or Ramelteon for delirium prevention in the intensive care unit: a systematic review and meta-analysis of randomized controlled trials.

PRISMA 2020 Checklist - Supplemental digital content

Section and Topic	Item #	Checklist item	Location where item is reported
Synthesis methods	13a	Describe the processes used to decide which studies were eligible for each synthesis (e.g. tabulating the study intervention characteristics and comparing against the planned groups for each synthesis (item #5)).	
	13b	Describe any methods required to prepare the data for presentation or synthesis, such as handling of missing summary statistics, or data conversions.	
	13c	Describe any methods used to tabulate or visually display results of individual studies and syntheses.	
	13d	Describe any methods used to synthesize results and provide a rationale for the choice(s). If meta-analysis was performed, describe the model(s), method(s) to identify the presence and extent of statistical heterogeneity, and software package(s) used.	
	13e	Describe any methods used to explore possible causes of heterogeneity among study results (e.g. subgroup analysis, meta-regression).	
	13f	Describe any sensitivity analyses conducted to assess robustness of the synthesized results.	Page 8-9
Reporting bias assessment	14	Describe any methods used to assess risk of bias due to missing results in a synthesis (arising from reporting biases).	Supplementary materials
Certainty assessment	15	Describe any methods used to assess certainty (or confidence) in the body of evidence for an outcome.	Page 6 –Figure 2
**RESULTS**			
Study selection	16a	Describe the results of the search and selection process, from the number of records identified in the search to the number of studies included in the review, ideally using a flow diagram.	Page 6 – supplementary materials
	16b	Cite studies that might appear to meet the inclusion criteria, but which were excluded, and explain why they were excluded.	
Study characteristics	17	Cite each included study and present its characteristics.	Table 1
Risk of bias in studies	18	Present assessments of risk of bias for each included study.	Supplementary materials
Results of individual studies	19	For all outcomes, present, for each study: (a) summary statistics for each group (where appropriate) and (b) an effect estimate and its precision (e.g. confidence/credible interval), ideally using structured tables or plots.	

Melatonin or Ramelteon for delirium prevention in the intensive care unit: a systematic review and meta-analysis of randomized controlled trials.

PRISMA 2020 Checklist - Supplemental digital content

Section and Topic	Item #	Checklist item	Location where item is reported
Results of syntheses	20a	For each synthesis, briefly summarise the characteristics and risk of bias among contributing studies.	
	20b	Present results of all statistical syntheses conducted. If meta-analysis was done, present for each the summary estimate and its precision (e.g. confidence/credible interval) and measures of statistical heterogeneity. If comparing groups, describe the direction of the effect.	Table 2
	20c	Present results of all investigations of possible causes of heterogeneity among study results.	
	20d	Present results of all sensitivity analyses conducted to assess the robustness of the synthesized results.	
Reporting biases	21	Present assessments of risk of bias due to missing results (arising from reporting biases) for each synthesis assessed.	
Certainty of evidence	22	Present assessments of certainty (or confidence) in the body of evidence for each outcome assessed.	
**DISCUSSION**			
Discussion	23a	Provide a general interpretation of the results in the context of other evidence.	Page 12-13-14
	23b	Discuss any limitations of the evidence included in the review.	Page 14-15
	23c	Discuss any limitations of the review processes used.	
	23d	Discuss implications of the results for practice, policy, and future research.	Page 15
**OTHER INFORMATION**			
Registration and protocol	24a	Provide registration information for the review, including register name and registration number, or state that the review was not registered.	Page 4
	24b	Indicate where the review protocol can be accessed, or state that a protocol was not prepared.	Page 4
	24c	Describe and explain any amendments to information provided at registration or in the protocol.	
Support	25	Describe sources of financial or non-financial support for the review, and the role of the funders or sponsors in the review.	
Competing interests	26	Declare any competing interests of review authors.	
Availability of data, code and other materials	27	Report which of the following are publicly available and where they can be found: template data collection forms; data extracted from included studies; data used for all analyses; analytic code; any other materials used in the review.	

Melatonin or Ramelteon for delirium prevention in the intensive care unit: a systematic review and meta-analysis of randomized controlled trials.

PRISMA 2020 Checklist - Supplemental digital content

From: Page MJ, McKenzie JE, Bossuyt PM, Boutron I, Hoffmann TC, Mulrow CD, et al. The PRISMA 2020 statement: an updated guideline for reporting systematic reviews. BMJ 2021;372:n71. doi: 10.1136/bmj.n71

For more information, visit: http://www.prisma-statement.org/
